# Construction of Protein-related Risk Score Model in Bladder Urothelial Carcinoma

**DOI:** 10.1155/2020/7147824

**Published:** 2020-07-31

**Authors:** Qizhan Luo, Xiaobo Zhang

**Affiliations:** ^1^Xiangya International Medical Center, Department of Geriatrics, Xiangya Hospital, Central South University, Changsha, China; ^2^Department of Urology, RWTH Aachen University, Pauwelsstrasse 30, 52072 Aachen, Germany

## Abstract

**Background:**

Though there are several prognostic models, there is no protein-related prognostic model. The aim of this study is to identify possible prognostic-related proteins in bladder urothelial carcinoma and to try to predict the prognosis of bladder urothelial carcinoma based on these proteins.

**Methods:**

Profile data and corresponding clinical traits were obtained from The Cancer Proteome Atlas (TCPA) and The Cancer Genome Atlas (TCGA) expression. Survival-associated protein in bladder urothelial carcinoma patients were estimated with Kaplan-Meier (KM) test and COX regression analysis. The potential molecular mechanisms and properties of these bladder urothelial carcinoma-specific proteins were also explored with the help of computational skills. The risk score model was validated in different clinical traits. Sankey diagram representation is for protein correlation. A new prognostic-related risk model based on proteins was developed by using multivariable COX analysis. Next, the alteration of the corresponding genes to the 6 prognostic-related proteins was analyzed. Finally, the relation between the corresponding genes and the immune infiltration was analyzed using the TIMER.

**Results:**

Six proteins were identified to be associated with the prognosis of bladder urothelial carcinoma. A prognostic signature based on proteins (BECLIN, EGFR, PKCALPHA, SRC, ANNEXIN1, and AXL) performed moderately in prognostic predictions. The alteration of corresponding genes was in 31(24%) sequenced cases. ANXA1, AXL, and EGFR were positively related to CD8+ T cell.

**Conclusion:**

Our results screened six proteins of clinical significance. The importance of a personalized protein signature model in the recognition, surveillance. The abnormal expression of six prognostic-related proteins may be caused by corresponding gene alteration. Furthermore, these proteins may affect survival via the immune infiltration.

## 1. Introduction

One of the most common urological carcinomas is bladder urothelial carcinoma. It is a complex biological mechanism. It is well known for its rapid metastasis to another part of the body also it has high recurrence rates [1]. The genesis of bladder urothelial carcinoma is highly associated with smoking, sex, age, schistosomiasis infection, and chemical contact [2, 3]. Current treatment and prognosis still heavily rely on clinical and pathologic staging that does not always reflect the individual condition of the patient.

The mechanism of bladder cancer is found as being a result of alterations in different molecular and pathways. Based on alterations in these molecular or pathways, different assessment of biomarkers that give novel insights into bladder urothelial carcinoma mechanism [4]. Therefore, future treatment and prognosis will add prognostic molecular models for risk stratification and management personalization.

TCGA and TCPA have a great advantage to help our understanding of the complex mechanism. There is no study that is a protein-related risk score model. We downloaded the data from both databases and developed a prognostic signature model based on bladder urothelial carcinoma proteins.

## 2. Method

### 2.1. The Protein Date BC from TCPA and the Clinical Data from TCGA Were Analysed

The clinical data of 409 bladder cases were obtained from TCGA dataset on the 9th of Feb 2020. The protein profiles of 344 samples were obtained from TCPA dataset on the 9th of Feb 2020. The clinical data was level 4. Because our data was downloaded directly from TCGA and TCPA databases, and we strictly observe the publishing guidelines provided by TCGA and TCPA; there was no requirement for ethical approvals.

### 2.2. Identification of Survival-Associated Proteins

Missing protein data was supplied and was performed with R software package “impute.” Survival time and survival status were extracted from the clinical characteristic which was corresponding to the expression data of proteins. To investigate the prognostic value of proteins in bladder urothelial carcinoma patients, single-factor Cox regression analysis and Kaplan-Meier analysis with the “survival” package and the *P* value <0.05. Kaplan-Meier analysis was performed based on the mediate of each protein expression. Patients have been divided into high-risk and low-risk groups. When the *P* value of single factor Cox regression analysis and Kaplan-Meier analysis both were <0.05, the significant prognostic-related proteins were obtained to build the protein-related prognosis model. Volcano plots were conducted with “dplyr,” “ggplot2,” and “ggrepel” R software to show significant proteins in the high risk and low-risk groups, two groups based on mediate of each protein expression.

### 2.3. Construction of a Prognostic Signature

The prognosis-related proteins were analyzed via the multifactor Cox regression model with R software “survival” package. Both direction selections were carried out based on the significant prognostic-related proteins. Kaplan-Meier survival curve was performed by “survival” and “survminer” package based on the level of highly optimal prognostic-related protein expression. Patients were assigned to high-risk and low-risk groups according to the median value of highly optimal prognostic-related protein expression. Risk scores were obtained based on the expression level of each highly optimal prognostic-related protein multiplied by its corresponding regression coefficient. Using the above methods, it has been calculated the risk scores of each case. Patients were assigned to high-risk and low-risk groups according to the median risk scores.

### 2.4. Validation of the Protein Signature

Kaplan-Meier survival curve was performed by “survival” and “survminer” package to compare the survival time of two groups. The risk curve was performed with the R software “heatmap” package to show the relationship between risk scores and survival time. Highly prognostic-related proteins were compared between two groups with Wilcox. test. It has been combined risk score with the available pathologic and the clinical traits in univariate and multivariate analysis to verify whether the risk score was an independent prognostic factor. The prognostic accuracy of risk score was measured using the AUC of the ROC curve with 0.5 indicting random chance and 1.0 indicating perfect classification. AUC of ROC curve was carried out by the R software package survival ROC. For further validation of the risk score model, survival time was compared between the high-risk group and low-risk group in different clinical traits.

### 2.5. Correlation between the Highly Prognostic-Related Proteins and Expression Protein

To explore the regulatory mechanisms of prognostic proteins, it has been chosen the highly prognostic-related proteins among the expression protein of BC patients. The correlation was analyzed using Pearson correlation analysis. CorFilter equals 0.4 and *P* value is <0.001. Sankeycharts were performed with R software ggplot2, ggalluvial, and dplyr package.

### 2.6. The Alteration of the Corresponding Genes to the 6 Prognostic-Related Proteins

We transferred the protein ID to gene ID. The corresponding genes to the 6 prognostic-related proteins were analyzed in the cBioPortal database. The alteration in selected genes was shown website (http://www.cbioportal.org/). The styles of alteration of genes also were analyzed in the cBioPortal database.

### 2.7. Correlations between the Corresponding Genes Expression and Immune Cells in TIMER

The corresponding genes to the 6 prognostic-related proteins were analyzed in TIMER. The relationship between the corresponding genes and the immune infiltration was analyzed using the TIMER (http://cistrome.org/TIMER/). CorFilter equals 0.3 and *P* value is <0.001. TIMER also draws Kaplan-Meier plots for immune infiltrates to visualize the survival differences.

## 3. Result

### 3.1. After Screening

**Figure 1:**
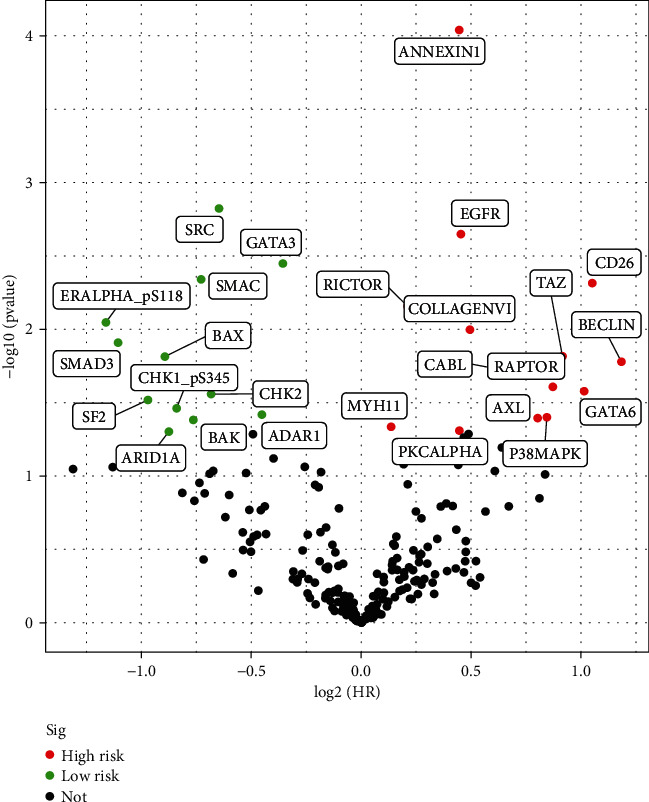
Volcano plots show significant prognostic proteins in the high-risk and low-risk groups.

17 proteins were significantly correlated to prognosis in BC. The names of proteins are BAK, BECLIN, EGFR, GATA3, PKCALPHA, SMAD3, SRC, ARID1A, RICTOR, SF2, TAZ, ANNEXIN1, ADAR1, SMAC, AXL, GATA6, and CABL (Table 1). Volcano plots show significant proteins in the high-risk and low-risk groups (Figure 1).

### 3.2. Construction of a Prognostic Signature

**Figure 2:**
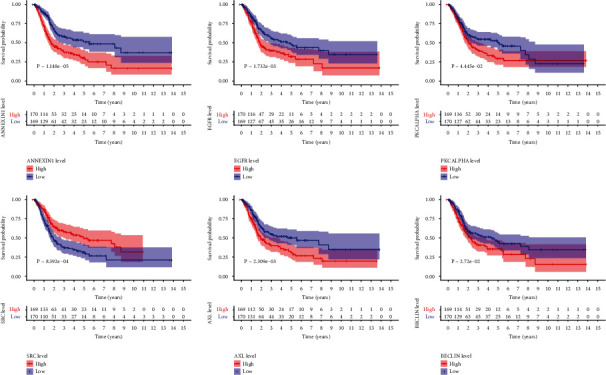
Kaplan-Meier survival curve among high-risk and low-risk groups based on the 6 highly optimal prognostic-related protein. Patients were assigned to high-risk and low-risk groups according to the median level of each highly optimal prognostic-related protein expression.

Both direction selections were carried out based on the 17 significant prognostic-related proteins, and 6 highly optimal prognostic-related proteins were found to be the final prognostic-related proteins (Table 2). Kaplan-Meier survival curve showed based on the 6 highly optimal prognostic-related protein expression (Figure 2). The risk score was calculated as the following formula: Risk scores = 1.285 × BECLIN + 0.286 × BGFR + 0.242 × PKCALPHA − 0.240 × SRC + 0.293 × ANNEXIN1 + 0.556 × AXL. Kaplan-Meier survival curve was performed by “survival” and “survminer” package with the highly optimal prognostic-related protein expression.

### 3.3. Validation of the Protein Signature

**Figure 3:**
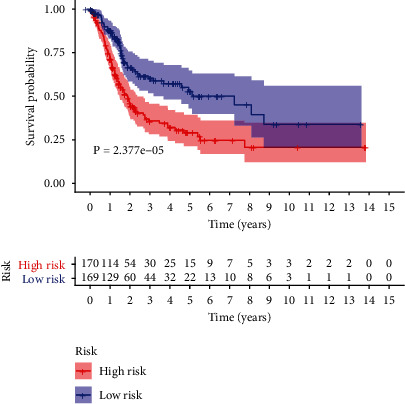
Kaplan-Meier survival curve among two groups based on risk score.

**Figure 4:**
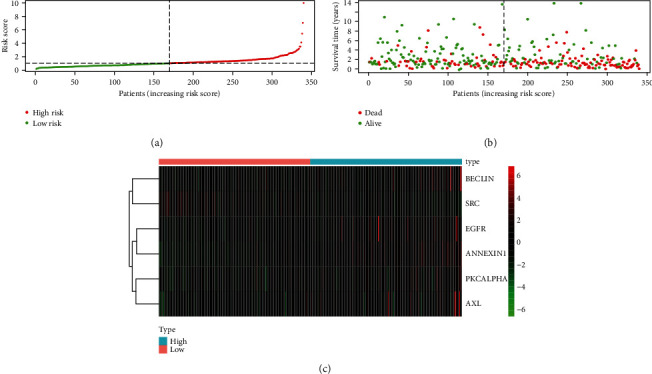
Risk curve shows the relationship between risk scores and survival time and status. (a). Rank of risk score and distribution of groups. (b). Survival status of patients in different groups. (c). Heatmap of expression profiles of included proteins.

**Figure 5:**
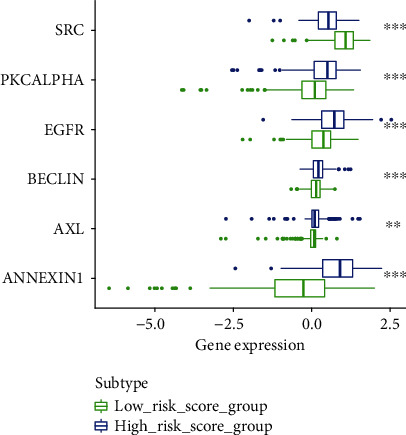
Highly prognostic-related proteins in low-risk score group and high-risk score group.

**Figure 6:**
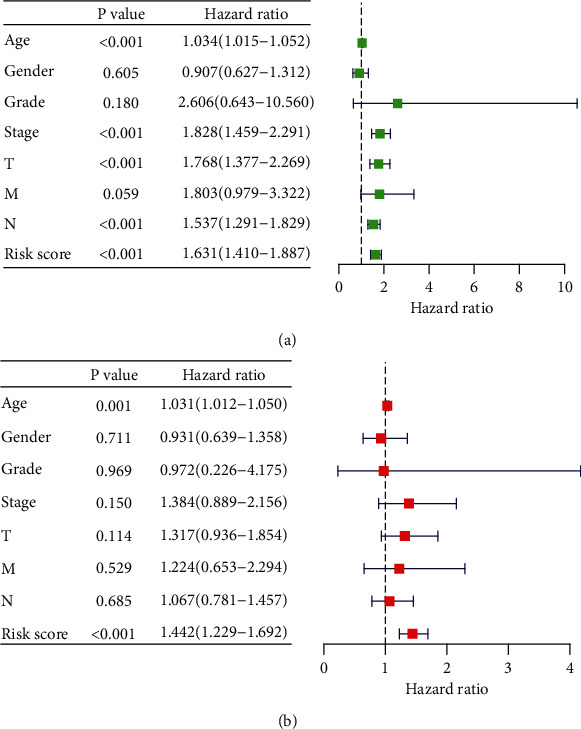
(a) Univariate analyses. (b) Multivariate analyses to verify risk scores were an independent prognostic factor.

**Figure 7:**
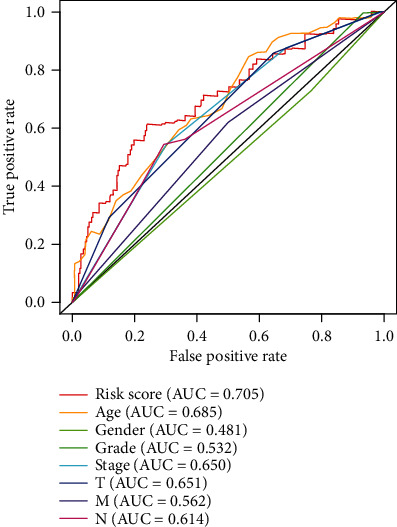
The prognostic accuracy of risk score.

**Figure 8:**
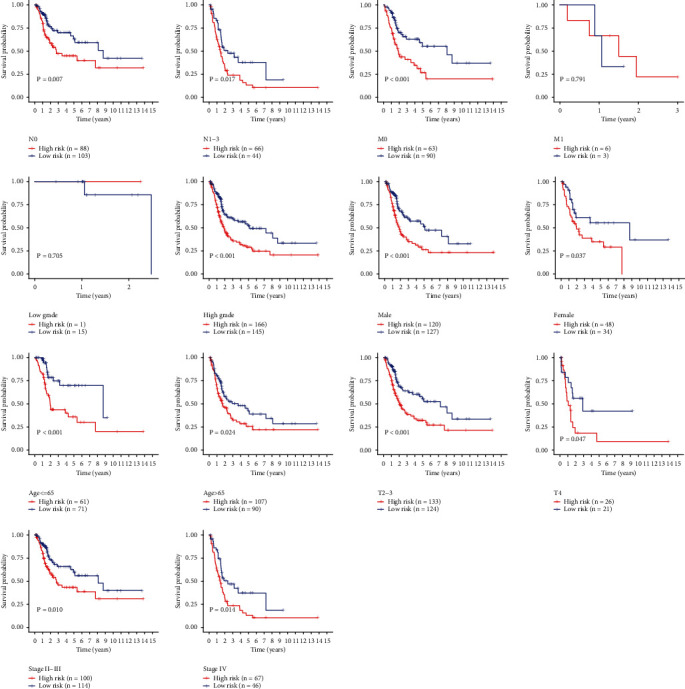
Survival time in high-risk score group and low-risk score group in different clinical traits.

Kaplan-Meier survival curve was performed to compare the survival time of the two groups based on the risk score (Figure 3). The risk curve was performed to show the relation between risk scores and survival rates (Figure 4). Highly prognostic-related proteins were compared between two groups (Figure 5). Three asterisks mean that *P* value is less than 0.001. Two asterisks mean that *P* value is less than 0.01. One asterisk means that *P* value is less than 0.05. Ns means that there is no significance difference between the two groups. It has been found the risk score was an independent prognostic factor (Figure 6). The prognostic accuracy of risk score was measured using the AUC of the ROC curve, which is 0.705 (Figure 7). Survival time was compared between high-risk score group and low-risk score group in different clinical traits (Figure 8).

### 3.4. Correlation between the Highly Prognostic-Related Proteins and Expression Protein

**Figure 9:**
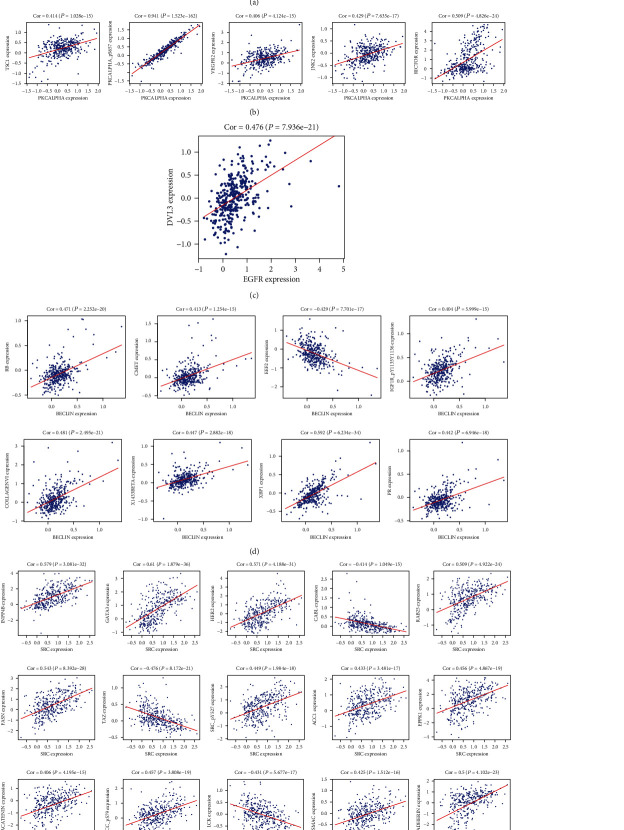
The correlation of protein. (a). Correlation between ANNEXIN1 and (FIBRONECTIN, PAI1, AR, GATA3). (b). Correlation between PKCALPHA and (PKCALPHA_pS657, VEGFR2, RICTOR, TSC1). (c). Correlation between EGFR and DVL3. (d). Correlation between BECLIN and (COLLAGENVI, PR, XBP1, X1433BETA, CMET, RB, IGF1R_pY1135Y1136, EEF2). (e). Correlation between SRC and (ACC_pS79, ACC1, BETACATENIN, ECADHERIN, GATA3, HER2, INPP4B, SRC_pY527, FASN, RAB25, EPPK1, SMAC, LCK, SRC, TAZ).

**Figure 10:**
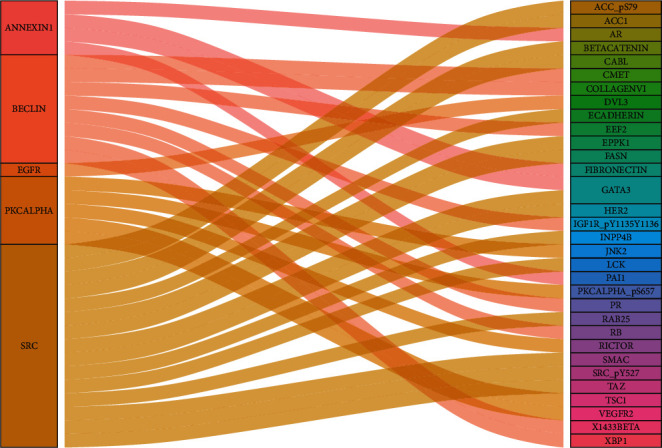
Sankeycharts were performed among highly optimal prognostic-related proteins and expression proteins.

The correlation was analyzed using Pearson's correlation analysis (Figure 9). Sankeycharts were performed (Figure 10). This regulatory network revealed the regulatory relationships among these proteins.

### 3.5. The Corresponding Genes to the 6 Prognostic-Related Proteins in the cBioPortal Database

**Figure 11:**
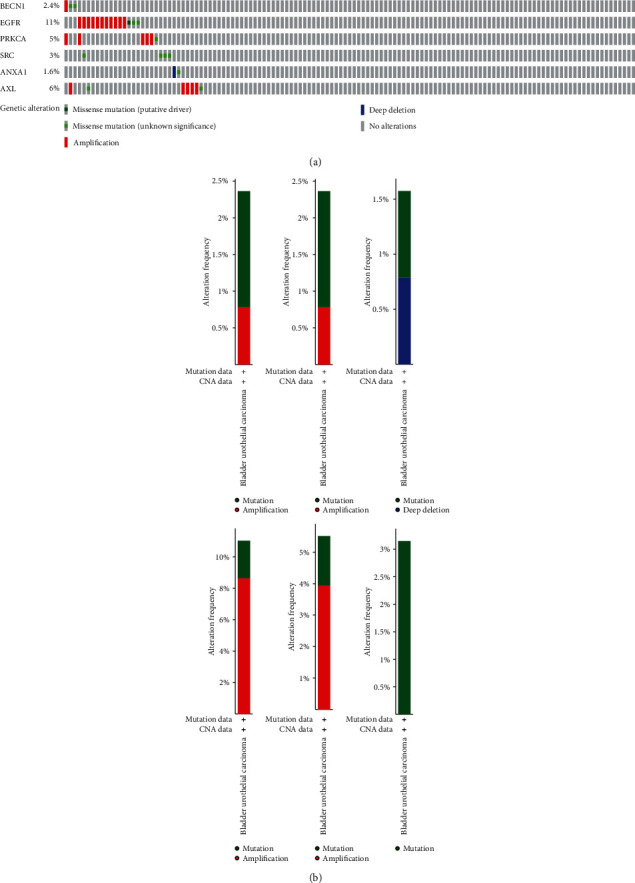
(a) The rate of 6 genes altered. B1.The types of alteration of BECN1 B2. The types of alteration of PRKCA. B3.The types of alteration of ANXA1. B4.The types of alteration of EGFR. B5.The types of alteration of AXL. B6.The types of alteration of SRC.

The corresponding genes to the 6 prognostic-related proteins were analyzed in the cBioPortal database. The rate of 6 genes altered in all cases as shown in Figure 11(a). The types of alteration of genes were shown in Figure 11(b).

### 3.6. Correlations between the Corresponding Genes Expression and Immune Cells in TIMER

**Figure 12:**
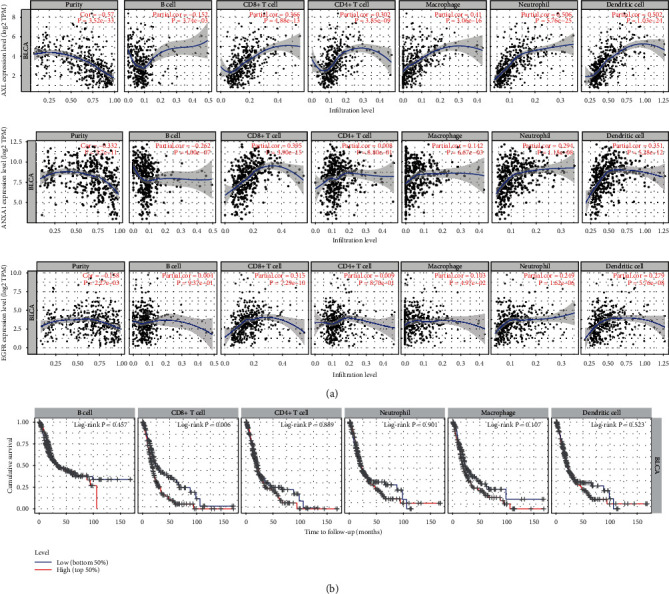
(a) The relationship between genes and immune infiltration. (b) TIMER drew Kaplan-Meier plots for CD8+ T cell.

The corresponding genes to the 6 prognostic-related proteins were analyzed in TIMER. The relationship between genes and immune infiltration was shown in Figure 12(a). TIMER drew Kaplan-Meier plots for CD8+ T cell to visualize the survival significantly differences in Figure 12(b).

## 4. Discussion

Careful assessing sensitive and novel biomarkers could monitor the progress and prognosis of carcinoma outcomes [5–11]. Based on alterations in these molecular or pathways, different assessment of biomarkers that give novel insights into bladder urothelial carcinoma mechanism. In an effort to bolster the clinical tool in bladder urothelial carcinoma, it has been developed a prognostic model to predict the prognosis in order to bolster the clinical tool in bladder carcinoma.

In the current study, we obtained seventeen significant prognostic candidate proteins and established a novel six-protein prognostic model. According to the further analysis, the gender, stage, T status, and M status had statistical significance in univariate analyses. These factors did not have statistical significance in multivariate analyses. However, the six-protein prognostic model had a statistical significance, which was proved to be an independent prognostic indicator (Figure 6). The six-protein expressions were significantly different in the two groups (Figure 5). Furthermore, the prognostic significance of the six genes was performed by multivariate analysis. EGFR, SRC, ANNEXIN1, and AXL were independent prognostic proteins (*P* = 0.012, 0.003, 0.004, and 0.041, respectively). The AUC of ROC curve was a moderate classification (Figure 7). In order to validate the accuracy of the risk score, the survival time was compared between high-risk score group and low-risk score group in different clinical traits (Figure 8). The risk score model was accurate except the risk model in M0 status and low grade. Because in M0 status and low grade, the cases were too little. The six-protein signature significantly stratified patients into high-risk and low-risk groups based on the intermediate value of risk score independent of clinical and pathologic factors. High-risk group had significantly poor prognosis (Figures 3 and 4). All the above results showed that the six-protein prognostic could play as an effective marker for bladder urothelial carcinoma prognosis prediction. This model could help clinicians make an accurate decision and avoid describing unnecessary medications and adverse drug effects, while it is strongly suggested other patients were high-risk to make treatment.

Kaplan-Meier survival curve among high-risk and low-risk groups based on the 6 highly optimal prognostic-related protein. Patients were divided into high risk and low-risk groups based on the median level of each high optimal prognostic-related protein expression. The survival time of high-risk and low-risk groups of each protein had statistical significance (Figure 2).

Since the correlation network among the protein is one of the most common and useful statistics, it has been elucidated the correlation and the mechanism of protein. To date, several studies have been published about PKCALPHA in bladder disease. Apart from the fact that the function of PKCALPHA is not clear. PRKCA is the gene, and the gene-synonym is PKC-alpha. PKC-alpha is one subtype of classical protein kinase C which is a candidate for PDK-2 in T cells on TCR stimulation [12]. In addition, it is a proinflammatory [13]. In the bladder, PKC-alpha is closely associated with the recurrence of bladder cancer [14]. PKC-alpha can promote proliferation, migration, and the survival rate of carcinoma cells via the downstream signal transduction pathways ERK1/2 and NF-*κ*B [15]. PKC alpha regulated the trin-1/UNC5B-mediated survival pathway in bladder cancer [16]. In the present study, the overexpression PKC-alpha was a high-risk factor in bladder urothelial carcinoma (Figures 1 and 2). PKC-alpha between PKCALPHA and PKCALPHA_pS657, VEGFR2, RICTOR, and TSC1 were positive correlations. However, there has been no publication about the correlation between the PKC-alpha, JNK2, RICTOR, and TSC-1 in bladder tumor (Figures 11 and 12).

Though PKC-alpha and RICTOR are in the mTOR signaling pathway [17–19] in the bladder tumor, it is uncertain that the PKC-alpha and RICTOR are the mTOR signaling pathway. Therefore, we suggest a lab experiment to verify it. The miR-200a overexpression causes low expression, which results in the upregulation of JNK2 expression and promotes the bladder cancer invasion [20]. Nevertheless, predicting the positive correlation between PKC-alpha and JNK2, still, there is no experimental study to prove this hypothesis. Furthermore, the function of PKC-alpha and JNK2 in the bladder tumor, we need further research to explore them.

Then, the alteration of the corresponding genes to the 6 prognostic-related proteins was analyzed in 127 Firehose legacy sample in the cBioPortal database. The alteration of genes in 31 (24%) sequenced cases was shown in Figure 11(a). The style of alteration of genes was different (Figure 11(b)). The alteration styles of BECN1, EGFR, PRKCA, and ALX were mutation and amplification. The alteration style of SRC was only the mutation. Moreover, the alteration styles of ANXA1 were mutation and deep deletion.

Finally, the corresponding genes to the 6 prognostic-related proteins were analyzed to reveal the correlation between genes and immune infiltration. ANXA1 is crucial to provide immunity as it helps CD8+ T cell stimulation dendritic cell present antigen [21]. Figure 12(a) shows the expression of ANXA1 is strongly and positively related to CD8+ T cell. Maybe ANXA1 had a similar function in bladder. A study reported that Axl knockout tumors had more infiltration of CD8+ T cells after radiation [22]. However, in the present study, AXL was strong and positively correlated to CD8+ T cell from Figure 12(a). CD8+ T cells expressing EGFR could benefit from EGFR ligands produced by the tumor [23]. EGFR is beneficial to CD8+ T cells in bladder cancer. From Figure 12(a), the expression of EGFR was strongly and positively related to CD8+ T cell. Figure 12(a) revealed TIMER drew Kaplan-Meier plots for CD8+ T cell to visualize the survival significantly differences in Figure 12(b). All the figures above suggested ANXA1, AXL, and EGFR may affect the prognosis via transcribing the proteins and medicating CD8+ T cell.

There are still some limitations in our research, and our data are downloaded from the analysis of publicly expressed data. We lack the lab or clinical experiment to verify these results. So, further research on these proteins will be the focus of our next step.

In this study, our results screened six proteins of clinical significance. The importance of a personalized protein signature model in the recognition, surveillance. The abnormal expression of six prognostic-related proteins may be caused by corresponding gene alteration. Furthermore, these proteins may affect survival via the immune infiltration.

## Figures and Tables

**Table 1:** 17 proteins were significatntly correlated to prognosis in BC.

Gene	KM	HR	HR.95 L	HR.95H	*P* value
ANNEXIN1	1.15E-05	1.362261548	1.166818065	1.590442059	9.13E-05
TAZ	0.00013013	1.886722131	1.12970026	3.151030876	0.015266466
SF2	0.000454652	0.510368312	0.2776481	0.938150894	0.030348532
BAK	0.000708446	0.588931975	0.353990253	0.979803449	0.041497671
SRC	0.000839191	0.638626026	0.484164999	0.842364074	0.001502422
GATA3	0.000860212	0.781102448	0.66153008	0.922287668	0.003565311
EGFR	0.001732026	1.369395712	1.119307856	1.6753609	0.002247458
ARID1A	0.001860228	0.545250791	0.297422122	0.999584103	0.049842967
AXL	0.002309345	1.744438807	1.024848166	2.969285454	0.040324605
GATA6	0.003401439	2.020152915	1.085751303	3.75870403	0.026441307
CABL	0.00431513	1.522389409	1.073197575	2.159592573	0.018474699
SMAC	0.014092504	0.603575018	0.425783275	0.855606182	0.004569768
RICTOR	0.01732931	1.157220752	1.040793571	1.286671927	0.00695473
BECLIN	0.027199215	2.272028814	1.160499759	4.448182682	0.016655813
ADAR1	0.03454304	0.731078894	0.543672655	0.983084848	0.038185369
SMAD3	0.036176387	0.464509845	0.254776118	0.846898045	0.0123406
PKCALPHA	0.044446818	1.362756846	1.001230588	1.854823697	0.049093018

**Table 2:** 6 highly optimal prognostic-related proteins and their coefficient.

Id	Coef	HR	HR.95L	HR.95H	*P* value
BECLIN	1.284587895	3.613178642	1.827081627	7.145307415	0.000222126
EGFR	0.286373326	1.331589479	1.055793909	1.679428652	0.015585541
PKCALPHA	0.242223245	1.274078593	0.949494745	1.70962111	0.106413498
SRC	-0.240328123	0.786369793	0.579236289	1.067573739	0.123376435
ANNEXIN1	0.293178172	1.340681641	1.122426065	1.60137698	0.001221101
AXL	0.555691215	1.743145457	1.01996076	2.979091162	0.042128919

## Data Availability

The data used to support the findings of this study are available from the corresponding author upon request.

## References

[1] Alfred Witjes J., Lebret T., Compérat E. M. (2017). Updated 2016 EAU guidelines on muscle-invasive and metastatic bladder cancer. *European Urology*.

[2] Letašiova S., Medveďová A., Šovčíková A. (2012). Bladder cancer, a review of the environmental risk factors. *Environmental Health*.

[3] Mostafa M. H., Sheweita S. A., O’Connor P. J. (1999). Relationship between schistosomiasis and bladder cancer. *Clinical Microbiology Reviews*.

[4] Mitra A. P., Daneshmand S., Daneshmand S., Chan K. (2018). Molecular Prognostication in Bladder Cancer. *Genitourinary Cancers. Cancer Treatment and Research*.

[5] Liao P., Li W., Liu R. (2018). Genome-scale analysis identifies SERPINE1 and SPARC as diagnostic and prognostic biomarkers in gastric cancer. *OncoTargets and Therapy*.

[6] Wang J. L., Yang M. Y., Xiao S., Sun B., Li Y. M., Yang L. Y. (2018). Downregulation of castor zinc finger 1 predicts poor prognosis and facilitates hepatocellular carcinoma progression via MAPK/ERK signaling. *Journal of Experimental & Clinical Cancer Research*.

[7] Li J., Yang Z., Zou Q. (2014). PKM2 and ACVR 1C are prognostic markers for poor prognosis of gallbladder cancer. *Clinical & Translational Oncology*.

[8] Ke M. J., Ji L. D., Li Y. X. (2019). Explore prognostic marker of colorectal cancer based on ceRNA network. *Journal of Cellular Biochemistry*.

[9] Dhruva Biswas, TRACERx Consortium, Birkbak N. J., Rosenthal R. (2019). A clonal expression biomarker associates with lung cancer mortality. *Nature Medicine*.

[10] Willenbrock F., Cox C., Wilhelm-Benartzic C. (2019). CCL5 is associated with poor prognosis in locally advanced pancreatic cancer (LAPC): biomarker analysis from the randomised phase II SCALOP trial. *Annals of Oncology*.

[11] Li G., Li M., Liang X. (2017). Identifying DCN and HSPD1 as Potential Biomarkers in Colon Cancer Using 2D-LC-MS/MS Combined with iTRAQ Technology. *Journal of Cancer*.

[12] Yang L., Qiao G., Ying H., Zhang J., Yin F. (2010). TCR-induced Akt serine 473 phosphorylation is regulated by protein kinase C-alpha. *Biochemical and Biophysical Research Communications*.

[13] Yang L. F., Kong H. M., Zhang X. Q., Yin F. (2015). Roles of PKCalpha on the biological functions of T cells. *Zhongguo Dang Dai Er Ke Za Zhi*.

[14] Kong C., Zhu Y., Liu D. (2005). Role of protein kinase C-alpha in superficial bladder carcinoma recurrence. *Urology*.

[15] Wu B., Zhou H., Hu L., Mu Y., Wu Y. (2013). Involvement of PKC*α* activation in TF/VIIa/PAR2-induced proliferation, migration, and survival of colon cancer cell SW620. *Tumour Biology*.

[16] Liu J., Kong C.-z., Gong D.-x., Zhang Z., Zhu Y.-y. (2014). PKC *α* regulates netrin-1/UNC5B-mediated survival pathway in bladder cancer. *BMC Cancer*.

[17] Meghan M., Young C. D., Wang S. (2015). Abstract B49: mTORC2 directs breast morphogenesis through Rictor-dependent PKC*α*/Rac1 signaling independent of Akt. *Molecular Cancer Therapeutics*.

[18] Chandrika G., Natesh K., Ranade D., Chugh A., Shastry P. (2016). Suppression of the invasive potential of Glioblastoma cells by mTOR inhibitors involves modulation of NF*κ*B and PKC-*α* signaling. *Scientific Reports*.

[19] Morrison M. M., Young C. D., Wang S. (2015). mTOR directs breast morphogenesis through the PKC-alpha-Rac1 signaling axis. *PLoS Genetics*.

[20] Yang R., Xu J., Hua X. (2020). Overexpressed miR-200a promotes bladder cancer invasion through direct regulating Dicer/miR-16/JNK2/MMP-2 axis. *Oncogene*.

[21] Sheikh M. H., Solito E. (2018). Annexin A1: Uncovering the Many Talents of an Old Protein. *International Journal of Molecular Sciences*.

[22] Aguilera T. A., Rafat M., Castellini L. (2016). Reprogramming the immunological microenvironment through radiation and targeting Axl. *Nature Communications*.

[23] Lozano T., Chocarro S., Martin C. (2019). Genetic modification of CD8^+^ T cells to express EGFR: potential application for adoptive T cell therapies. *Frontiers in Immunology*.

