# Corrigendum to “Construction of Protein-related Risk Score Model in Bladder Urothelial Carcinoma”

**DOI:** 10.1155/2021/9758361

**Published:** 2021-06-02

**Authors:** Qizhan Luo, Xiaobo Zhang

**Affiliations:** ^1^Xiangya International Medical Center, Department of Geriatrics, Xiangya Hospital, Central South University, Changsha, China; ^2^Department of Urology, RWTH Aachen University, Pauwelsstrasse 30, 52074 Aachen, Germany

In the article titled “Construction of Protein-related Risk Score Model in Bladder Urothelial Carcinoma” [1], the Acknowledgments section should read as follows:
